# Failure of Surgical Aortic Valve Prostheses: An Analysis of Heart Team Decisions and Postoperative Outcomes

**DOI:** 10.3390/jcm13154461

**Published:** 2024-07-30

**Authors:** Philipp Schnackenburg, Shekhar Saha, Ahmad Ali, Konstanze Maria Horke, Joscha Buech, Christoph S. Mueller, Sebastian Sadoni, Martin Orban, Rainer Kaiser, Philipp Maximilian Doldi, Konstantinos Rizas, Steffen Massberg, Christian Hagl, Dominik Joskowiak

**Affiliations:** 1 Department of Cardiac Surgery, LMU University Hospital, Marchioninistrasse 15, 81377 Munich, Germanysebastian.sadoni@med.uni-muenchen.de (S.S.);; 2 German Centre for Cardiovascular Research (DZHK), Partner site Munich Heart Alliance, 80802 Munich, Germany; 3 Department of Cardiology, LMU University Hospital and German Centre for Cardiovascular Research (DZHK), Partner Site Munich Heart Alliance, Marchioninistrasse 15, 81377 Munich, Germany

**Keywords:** Failed SAVR, ViV TAVR, Redo SAVR

## Abstract

**Objectives:** To analyze Heart Team decisions and outcomes following failure of surgical aortic valve replacement (SAVR) prostheses. **Methods:** Patients undergoing re-operations following index SAVR (Redo-SAVR) and those undergoing valve-in-valve transcatheter aortic valve replacement (ViV-TAVR) following SAVR were included in this study. Patients who underwent index SAVR and/or Redo-SAVR for endocarditis were excluded. Data are presented as medians and 25th–75th percentiles, or absolute numbers and percentages. Outcomes were analyzed in accordance to the VARC-3 criteria. **Results:** Between 01/2015 and 03/2021, 53 patients underwent Redo-SAVR, 103 patients ViV-TAVR. Mean EuroSCORE II was 5.7% (3.5–8.5) in the Redo-SAVR group and 9.2% (5.4–13.6) in the ViV group. In the Redo-SAVR group, 12 patients received aortic root enlargement (22.6%). Length of hospital and ICU stay was longer in the Redo-SAVR group (*p* < 0.001; *p* < 0.001), PGmax and PGmean were lower in the Redo-SAVR group as compared to the ViV-TAVR group (18 mmHg (10–30) vs. 26 mmHg (19–38), *p* < 0.001) (9 mmHg (6–15) vs. 15 mmHg (9–21), *p* < 0.001). A higher rate of paravalvular leakage was seen in the ViV-TAVR group (*p* = 0.013). VARC-3 Early Safety were comparable between the two populations (*p* = 0.343). Survival at 1 year and 5 years was 82% and 36% in the ViV-TAVR cohort and 84% and 77% in the Redo-SAVR cohort. The variables were patient age (OR 1.061; [95% CI 1.020–1.104], *p* = 0.004), coronary heart disease (OR 2.648; [95% CI 1.160–6.048], *p* = 0.021), and chronic renal insufficiency (OR 2.711; [95% CI 1.160–6.048], *p* = 0.021) showed a significant correlation to ViV-TAVR. **Conclusions:** Heart Team decisions are crucial in the treatment of patients with degenerated aortic bioprostheses and lead to a low mortality in both treatment paths thanks to patient-specific therapy planning. ViV-TAVR offers a treatment for elderly or intermediate-risk profile patients with comparable short-term mortality. However, this therapy is associated with increased pressure gradients and a high prevalence of paravalvular leakage. Redo-SAVR enables the surgical treatment of concomitant cardiac pathologies and allows anticipation for later VIV-TAVR by implanting the largest possible valve prostheses.

## 1. Introduction

Interdisciplinary Heart Teams have evolved considerably over the last decade and play a central role in the treatment of a wide range of cardiovascular diseases. In order to meet the individual needs of each patient in the face of constantly evolving therapeutic options, continuous self-reflection on the therapeutic decisions made and the results achieved is essential. This applies in particular to the management of aortic valve disease, which has changed dramatically over the last years. Improvement of transcatheter aortic valve replacement (TAVR) has offered an alternative strategy for treating degenerated biological surgical aortic valve prostheses [1]. Recent data have also shown that this therapy procedure is non-inferior to surgery in low- and intermediate-risk patients [2]. Valve-in-valve transcatheter aortic valve replacement (ViV-TAVR) has emerged in recent years as a novel alternative approach to conventional Redo surgical aortic valve replacement (Redo-SAVR) resulting in an increasing trend toward implantation of biological valves at primary surgical aortic valve replacement (SAVR), even in younger patients. The rationale is that ViV-TAVR can be performed with a lower perioperative risk compared to redo-SAVR in cases of prosthetic aortic valve dysfunction and therefore increasingly eliminating the need for mechanical heart valves with a high risk for thromboembolism and lifelong use of vitamin K antagonists [3,4,5]. This increases the demands on the Heart Team, which faces lifelong therapy decisions for patients with heart valve disease although hemodynamic outcomes are difficult to predict due to multiple factors including surgical valve size, surgical valve type, patient–prosthesis mismatch, and type of transcatheter valve [6]. Data with regard to hemodynamic performance and long-term outcomes are sparse. In this study, we endeavor to analyze Heart Team decisions and outcomes following the failure of surgical aortic valve prostheses at our institution.

## 2. Methods

### 2.1. Ethics Statement

This study was approved by the institutional ethics committee of the Ludwig Maximilian University (No. 21-0423), and informed consent was obtained in case of prospective follow-up data collection. Postoperative treatment and data collection were performed as a part of routine patient care. Data acquisition was based on institutional databases and was subsequently anonymized. All procedures described in this study were in accordance with the institutional ethics boards and national data safety regulations.

### 2.2. Study Design

A total of 156 patients who underwent either Redo-SAVR (n = 53) or ViV-TAVR (n = 103) after Index-SAVR with subsequent prosthesis dysfunction at our institution from January 2015 to March 2021 were included. Patients with a history of native or prosthetic valve endocarditis were excluded. Patients included in the study were retrospectively reviewed in accordance with national data safety regulations. All patients were discussed in the Heart Team and all patients were evaluated individually. Patient details were collected from institutional databases and surgeon notes and were anonymized for further analysis. Follow-up was achieved by routine check-ups and patient interviews. The European System for Cardiac Operative Risk Evaluation II (EuroSCORE II) and Society of Thoracic Surgeons Predicted Risk of Mortality (STS PROM) were used to predict the risk of perioperative mortality at the time of VIV-TAVR and Redo-SAVR. Endpoints were determined in accordance with the recommendations of the Valve Academic Research Consortium 3 (VARC-3) [7]. All patients were discharged to either cardiac rehabilitation centers, transferred to secondary care centers or discharged home.

### 2.3. Statistical Analysis

Data were analyzed using the IBM SPSS Statistics Data Editor^®^ version 25 (IBM Corp. Released 2017. IBM SPSS Statistics, Version 25.0. Armonk, NY, USA: IBM Corp.). Data were tested for normal distribution using the Kolmogorov–Smirnov test with Lillefors correction. Categorical variables were evaluated using the Chi-Squared and Fisher’s exact method and continuous variables were evaluated using the Mann–Whitney U test. Multivariable analysis incorporated logistic regression using a forward stepwise (conditional) model, where significance for entry was set at *p* < 0.05 and significance for exit was *p* < 0.10. The regression model was verified using the regression diagnostics which included the goodness-of-fit test as well as tests for autocorrelation, multicollinearity, and heteroscedasticity. Survival analysis was performed with the Kaplan–Meier curve. Illustrations were prepared using GraphPad Prism (Version 10, GraphPad Software Inc., San Diego, CA, USA). Data are presented as medians (25–75th quartiles) or as absolute numbers (percentages) unless otherwise specified.

## 3. Results

### 3.1. Patient Characteristics and Co-Morbidities

Patient characteristics and co-morbidities are listed in Table 1. Patients in the ViV cohort were significantly older (79 (75–83) years vs. 68 (59–77) years, *p* < 0.001) and had a significantly higher median EuroSCORE II (9.2 (5.4–13.6) vs. 5.7 (3.5–8.5), *p* < 0.001) and STS PROM score (4.1 (2.6–6.8) vs. (2.0 (1.3–2.8), *p* < 0.001) compared to the Redo-SAVR cohort. In the Redo-SAVR cohort, index SAVR was performed as a biological valve replacement in 81.1% of cases, and as a mechanical valve replacement in 18.8% of cases. Concomitant procedures were performed in 33.9% of cases. In the ViV-TAVR cohort concomitant procedures were performed in 48.5% of cases. In the Redo-SAVR cohort, 9 (5–14.5) years, and in the ViV-TAVR cohort 10 (7–12) years elapsed until secondary valve intervention. The most common indication for repeat valve replacement in the Redo-SAVR cohort was aortic valve stenosis (AS) (23 (43.4%)), and in the ViV-TAVR cohort, mixed aortic valve disease with predominant aortic stenosis (MAVD) (43 (41.7%)).

The ViV-TAVR cohort had a significantly higher rate of co-morbidities such as arterial hypertension (99 (96.1%) vs. 43 (81.1%), *p* = 0.005), hyperuricemia (26 (25.2%) vs. 5 (9.4%), *p* = 0.020), and insulin-dependent diabetes mellitus (22 (21.4%) vs. 3 (5.7%), *p* = 0.011). The ViV-TAVR cohort had significantly higher rates of coronary artery disease (71 (68.9%) vs. 17 (32.1%), *p* < 0.001), preoperative coronary angioplasty or stenting (28 (27.2%) vs. 6 (11.3%), *p* = 0.025). Regarding other co-morbidities, the ViV-TAVR cohort had significantly higher rates of chronic renal failure (56 (54.4%) vs. 11 (20.8%), *p* < 0.001) and significantly higher use of new oral anticoagulants (NOAKs) (31 (30.1%) vs. 5 (9.4%), *p* = 0.004). In contrast, patients in the Redo-SAVR cohort had significantly higher use of vitamin K antagonists (VKAs) ((17 (32.1%)) vs. (10 (9.7%), *p* = 0.001).

### 3.2. Heart Team Decision

To identify variables that strongly influenced Heart Team decisions, we performed a multivariate analysis (Table 2). Only those variables that showed a significant difference between the cohorts in the descriptive statistics were examined. The variables were patient age (OR 1.061; [95% CI 1.020–1.104], *p* = 0.004), coronary heart disease (OR 2.648; [95% CI 1.160–6.048], *p* = 0.021), and chronic renal insufficiency (OR 2.711; [95% CI 1.160–6.048], *p* = 0.021) showed a significant correlation to ViV-TAVR. The use of vitamin K antagonists showed a significant correlation to Redo-SAVR (OR 0.311; [95% CI 0.107–0.906], *p* = 0.032). There was no significant association with insulin-dependent diabetes mellitus and the use of NOACs.

### 3.3. Echocardiographic Data

**Figure 1:**
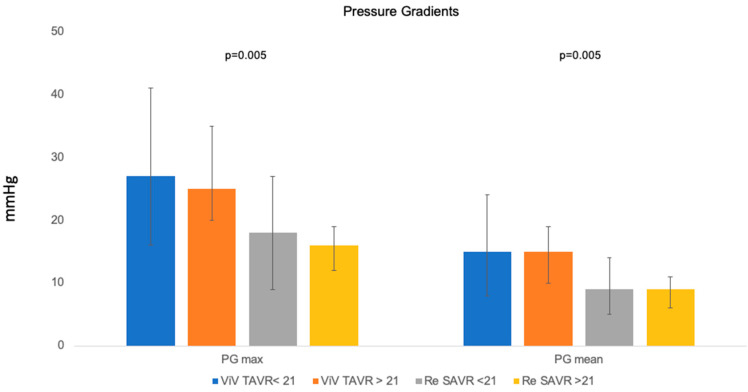
Pressure gradients.

Echocardiographic data are listed in Table 3. Before secondary intervention, the ViV-TAVR cohort had a significantly higher incidence of severe aortic valve stenosis (*p* = 0.010). There were no significant differences between cohorts regarding other valve diseases such as aortic regurgitation, mitral regurgitation, tricuspid regurgitation, and LVEF. After secondary intervention, the ViV-TAVR cohort had significantly higher maximum (26 (19–38) mmHg vs. 18 (10–30) mmHg, *p* < 0.001) and mean (15 (9–21) mmHg vs. 9 (6–15) mmHg, *p* < 0.001) gradients across the aortic valve prosthesis (Figure 1). In the ViV-TAVR cohort, prosthesis gradients above 20 mmHg were also significantly more frequent (25 (24.2%) vs. 1 (1.8%), *p* < 0.001), as well as PVL (23 (25.3%) vs. 1 (1.9%), *p* = 0.013).

### 3.4. Surgical Data

Details of surgery/intervention are listed in Table 4. In the Redo-SAVR cohort, median bypass time was 156 (119–211) minutes and cross-clamp time was 103 (82–141) minutes. Concomitant procedures were performed more frequently in patients in the Redo-SAVR cohort (33 (62.2%) versus 16 (15.5%)). The most common additional procedure performed was aortic root enlargement (22.6%), followed by aortic root replacement (5.7%), aortocoronary bypass (13.2%), mitral valve replacement (24.5%), tricuspid valve repair (3.8%), and mitral valve repair (1.9%). In the ViV-TAVR cohort, 16.5% of patients underwent additional PTCA or stenting during the procedure. In the Redo-SAVR cohort, 75.4% of patients underwent biological valve replacement and 24.5% underwent mechanical valve replacement.

### 3.5. Valve Type-Based Echocardiographic Data

Echocardiographic data are listed in Table 5a,b. There were no significant differences between bioprosthetic and mechanical valves in the RS group with regard to pressure gradients and paravalvular leakage. In the ViV cohort, patients with a self-expandable prosthesis had a significantly higher incidence of paravalvular leakage compared to those with a balloon-expandable prosthesis. We observed no differences with regard to the pressure gradients.

### 3.6. Postprocedural Morbidities and Outcomes

Postprocedural morbidities are listed in Table 6. Patients after Redo-SAVR had a significantly higher rate of re-explorative surgery (6 (11.3) vs. 0 (0), *p* = 0.001) and had a significantly higher number of packed red blood cells transfused (38 (71.6%) vs. 5 (4.8%), *p* < 0.001). Furthermore, patients in the Redo-SAVR group had a significantly higher rate of surgical site infections (5 (9.4%) vs. 1 (1.0%), *p* = 0.018). Cerebrovascular events occurred more frequently in patients after ViV-TAVR, without statistical significance between cohorts (5 (4.9%) vs. 0 (0%), *p* = 0.167). Patients in the Redo-SAVR group had a significantly higher rate of renal replacement therapy after valve replacement (7 (13.2%) vs. 2 (1.9%), *p* = 0.008).

Postprocedural outcomes are listed in Table 7. Patients after RS had a significantly longer stay at the ICU (3 (3–5) days vs. 1 (1–3) days, *p* < 0.001) and a significantly longer overall length of stay at the hospital (15 (13–20) days vs. 11 (8–14) days, *p* < 0.001). Patients after ViV-TAVR achieved VARC-3 Early Safety endpoint more often, without statistical significance between cohorts (43 (87.3%) vs. 90 (81.1%), *p* = 0.343). The mortality at 30 days was higher in the Redo-SAVR cohort, without statistical significance (3(5.7%) vs. 3 (2.9%), *p* = 0.409) as well.

Mean follow-up in the Redo-SAVR cohort was 2258 ± 101 days in the ViV-TAVR cohort 1937 ± 82 days (*p* = 0.008). At follow-up, survival at 1 year and 5 years was 82% and 36% in the ViV-TAVR cohort, and 84% and 77% in the Redo-SAVR cohort, respectively (*p* = 0.007).

Postprocedural morbidities and outcomes after the exclusion of Redo-SAVR patients with concomitant procedures are listed in Table 8. Patients after isolated Redo-SAVR had a significantly higher rate of atrial fibrillation (5 (17.2%) vs. 5 (4.9%), *p* = 0.041) and a significantly higher number of packed red blood cells transfused (18 (62.1%) vs. 5 (4.8%), *p* < 0.001). There was no significant difference with regard to reexplorative surgery, surgical site infections, and the need for renal replacement therapy.

## 4. Discussion

Following degeneration of aortic bioprostheses, treatment decisions should be made in an interdisciplinary Heart Team. This is important to ensure a patient-centric treatment plan with low mortality and morbidity as well as lifelong management of valvular heart disease [8,9]. The principal findings of the present study investigating the outcomes of Redo-SAVR and ViV-TAVR in the setting of SAVR prostheses failure may be summarized as follows:

- Redo-SAVR, being the more invasive therapy option, enables the surgical treatment of concomitant cardiac pathologies, and allows anticipation for later VIV-TAVR by implanting the largest possible valve prostheses or implantation of mechanical prostheses if mandatory.
- ViV-TAVR offers a treatment for elderly or intermediate-risk profile patients with comparable short-term mortality. However, this therapy is associated with increased pressure gradients and a high prevalence of paravalvular leakage.

### 4.1. Surgery or Intervention—The Heart Team Approach

The combined expertise of a Heart Team provides the basis for a more balanced appraisal of a specific case [10]. A Heart Team usually consists of cardiologists, cardiac surgeons, interventionists, imaging specialists, anesthesiologists, and mid-level providers. Furthermore, in some cases, the expert opinion of a general practitioner, geriatrician, or intensive care specialist can be of additional value [10]. Heart Teams have shown to be beneficial for patients, improving survival, and avoiding complications [10,11]. Advanced age alone should not be the main reason for intervention. Previous reports have shown that complex valve surgery may be carried out in elderly patients with good results [12,13]. Furthermore, a surgical approach offers the opportunity to therapeutically address concomitant cardiac disorders that occur in up to 30% of patients with degenerated prosthetic valves such as coronary artery disease, aortic aneurysms, hypertrophic cardiomyopathy, atrial fibrillation, as well as mitral and tricuspid valve dysfunction [14,15]. Surgery should not be immediately disregarded as a therapeutic option and should be carefully considered with a multidisciplinary approach in the Heart Team. Of vital importance is the planning of the index procedure and lifetime management of aortic valve disease. In young patients with calcific aortic valve disease, the Heart Team should plan ahead and take into consideration the need for Redo-SAVR surgery or re-interventions in the future [8,16]. At our institution, all cases are discussed in the Heart Team, and treatment options are discussed and implemented in a patient-centric, multidisciplinary fashion. This is reflected by advanced age, higher EuroSCORE II, and STS PROM in the ViV group as well as age, coronary artery disease, and chronic kidney insufficiency being associated with ViV-TAVR in the multivariate analysis.

### 4.2. Hemodynamic Outcomes

Since the advent of transcatheter technology, there has been an increase in the number of biological aortic valve prostheses implanted in comparison to mechanical valves, as they provide the possibility of valve re-intervention in the future. Furthermore, it has been shown that the size of valves implanted has also reduced over the years [17,18]. This is potentially dangerous at the time of reintervention, as small biological aortic valve prostheses at the index operation have been reported to be associated with a much higher 1-year mortality compared with intermediate- or large-sized biological aortic valve prostheses [18]. In the ViV-TAVR cohort, higher postinterventional maximum and mean gradients as well as higher rates of PVL were observed. It has been demonstrated that postoperative elevated gradients and PVL are not only clinically relevant but also contribute significantly to structural and nonstructural prosthetic valve degeneration [19,20]. Furthermore, other studies have reported a higher rate of aortic insufficiency in patients undergoing ViV-TAVR [21]. Studies have also shown an association between increased rehospitalization rates due to decompensated heart failure, the need for valve reintervention, and increased mortality in patients undergoing ViV-TAVR [22,23,24]. Data from the VIVID registry reports a severe prosthesis–patient mismatch in 31.8% of patients [25]. Therefore, the Heart Team often has to decide whether a patient who is actually classified for ViV-TAVR due to their age and risk profile should opt for Redo-SAVR with concomitant aortic root enlargement due to the risk of relevant PPM. According to our data, for patients at increased risk for poor hemodynamic outcome due to a small prosthesis size (<21 mm) or preexisting PPM, Redo-SAVR appears to be the superior therapy, even in cases of higher age and risk constellation, although current ESC/EACTS guidelines do not clearly recommend this [26].

### 4.3. Outcomes

Patients in the Redo-SAVR cohort had a significantly higher incidence of postoperative atrial fibrillation, perioperative blood transfusion, re-explorative surgery, surgical site infection, requirement of postoperative renal replacement therapy, and had significantly longer ICU and hospital stays. However, we observed no differences in the rates of myocardial infarction, new pacemaker implantation, hospital admission for cardiac reasons, major bleeding, and ischemic stroke among the groups. Despite higher invasiveness with concomitant higher surgical trauma and use of cardiopulmonary bypass, there was no difference in the rates of the VARC-3 Early Safety index. Another point that should be mentioned in this context is the high rate of concomitant procedures in the Redo-SAVR group. Exclusion of these patients shows a significant reduction in postoperative morbidities so significant differences were only found with regard to a higher rate of atrial fibrillation and a higher number of packed red blood cells transfused. Studies have shown that coronary artery obstruction occurs with an incidence of up to 3.5% during ViV-TAVR and is approximately four to six times more common compared to TAVR in native aortic valves [27]. In the ViV group, half of the cases of peri-interventional cardiopulmonary resuscitation and all cases of peri-interventional myocardial infarction were associated with coronary artery obstruction. Only one patient survived such an event. Despite advances in peri-interventional imaging, morphologic planning, and new interventional strategies, this remains a life-threatening complication that is absent with controlled removal of the degenerated prosthetic valve under visualization during Redo-SAVR [27].

In our study, the Redo-SAVR cohort had a comparable 30-day mortality to the ViV-TAVR cohort, with our results being comparable to the VIVID registry [25]. It should be noted that in the Redo-SAVR cohort, concomitant procedures were performed in more than 60% of cases, further increasing the perioperative risk due to higher invasiveness and longer cross-clamp/bypass time, and therefore biasing the comparison with interventional single-valve replacement. This should be carefully considered while interpreting the 30-day mortality. In the Redo-SAVR group, almost two-thirds of the patients underwent concomitant surgery. Although these enable holistic treatment of various cardiac pathologies, they are associated with a higher perioperative risk due to their greater complexity and invasiveness. If patients with combined procedures are excluded and thus only the isolated replacement of the valve prosthesis is considered, Patel et al. [21] reported a similar early mortality in the ViV cohort. Although the exclusion of combined interventions does not do justice to the clinical reality, it does show how important it is to consider age and risk distribution as well as the scope and complexity of the respective therapy in the assessment of mortality, especially in the short-term follow-up. In summary, the 30-day mortality in the ViV-TAVR cohort is likely to be lower in patients with a high-risk profile, but Redo-SAVR is a safe procedure with a comparable overall mortality that is due to new developments such as rapid deployment aortic valve prostheses with shorter implantation and thus operation times. However, there is further potential for improvement.

**Figure 2:**
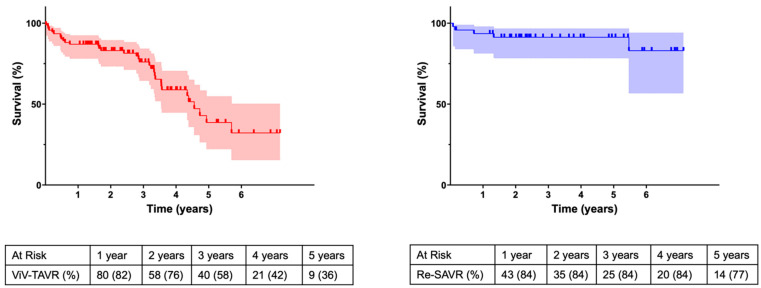
Kaplan–Meier survival curve.

The VIVID registry reports an overall 1-year survival of 83.2% and this is comparable to our results in both the groups (Figure 2). Although associated with an initial comparable or higher mortality, several other studies have also shown an improved long-term survival rate in patients undergoing Redo-SAVR [1,14,21]. Deharo et al. [1] showed that an initial mortality advantage of the ViV cohort was lost after a median of 516 days whereas Sedeek et al. [14] reported a similar reversal of the survival curve after approximately 2 years. Whether the reduced long-term survival of the ViV cohort is due to the age of the patients or the significantly increased prosthesis gradients cannot be conclusively clarified within the scope of this analysis.

Careful selection of patients in the Heart Team is critical to provide optimal therapy to each individual patient. Good surgery in primary or even secondary aortic valve replacement with the avoidance of small prosthesis sizes by augmentation of the aortic root allows for the reasonable and safe use of ViV-TAVR later, when open surgery is not an option because of the risk constellation or patient age.

## 5. Conclusions

Heart Team decisions are crucial in the treatment of patients with degenerated aortic bioprostheses and lead to a low mortality in both treatment paths due to patient-specific therapy planning. Redo-SAVR is associated with low morbidity and mortality and allows surgical repair of multiple cardiac diseases; ViV-TAVR offers a treatment alternative with comparable morbidity and mortality for the elderly or patients with an intermediate-risk profile. However, this therapy is associated with significantly higher transvalvular pressure gradients and a high prevalence of PVL compared to conventional Redo-SAVR. Foresighted surgery for primary or even secondary aortic valve replacement with holistic management of concomitant cardiac disease and the avoidance of small prosthesis sizes by aortic root augmentation allows for the reasonable and safe use of ViV-TAVR later, when open surgery is not an option because of the risk constellation or patient age.

### Limitations

This is a retrospective single-center study with the inherent limitations of such an analysis. The small number of patients is associated with a low power of statistical analyses. Further multicenter studies are required. Furthermore, there are obvious differences in the risk scores. Owing to the small sample size, propensity score matching and subgroup analyses based on valve type could not be performed.

## Figures and Tables

**Table 1:** Patient characteristics and co-morbidities.

	RS (n = 53)	ViV (n = 103)	*p*-Value
Age (years)	68 (59–77)	79 (75–83)	<0.001
Female	23 (43.4)	44 (42.7)	1.000
Body Mass Index (kg/m^2^)	25.3 (22.9–28.9)	25.3 (23.3–28.5)	0.855
EuroSCORE II	5.7 (3.5–8.5)	9.2 (5.4–13.6)	<0.001
STS—PROM	2.0 (1.3–2.8)	4.1 (2.6–6.8)	<0.001
Index surgery (%)			
Biological prosthesis	43 (81.1)	103 (100)	-
Mechanical prosthesis	10 (18.8)	-	-
Concomitant procedure	18 (33.9)	50 (48.5)	
Time to Redo-surgery (years)	9 (5–14.5)	10 (7–12)	0.978
Indication for Redo-surgery (%)			0.688
AS	23 (43.4)	38 (36.9)	-
AR	7 (13.2)	5 (4.9)	-
MAVD—predominant AS	10 (18.9)	43 (41.7)	-
MAVD—predominant AR	9 (17.0)	17 (16.5)	-
PVL	4 (7.5)	0 (0.0)	-
Co-morbidities			
Arterial hypertension (%)	43 (81.1)	99 (96.1)	0.005
IDDM (%)	3 (5.7)	22 (21.4)	0.011
Hyperlipoproteinemia (%)	38 (71.7)	85 (82.5)	0.182
Hyperuricemia (%)	5 (9.4)	26 (25.2)	0.020
Atrial fibrillation (%)	20 (37.7)	42 (40.8)	0.733
CAD (%)	17 (32.1)	71 (68.9)	<0.001
Previous PTCA/Stenting (%)	6 (11.3)	28 (27.2)	0.025
Previous MI (<90 days) (%)	1 (1.8)	5 (4.8)	0.665
Previous pacemaker implantation (%)	5 (9.4)	17 (16.5)	0.431
COPD (%)	5 (9.4)	10 (9.7)	1.000
PAD (%)	2 (3.8)	9 (8.7)	0.335
CVD (%)	9 (17.0)	29 (28.2)	0.168
Previous stroke (%)	2 (3.8)	14 (13.6)	0.091
CKD (%)	11 (20.8)	56 (54.4)	<0.001
Hemodialysis (%)	0 (0.0)	6 (5.8)	0.096
ECMO preoperative (%)	0 (0.0)	1 (1.0)	1.000
Anticoagulation			
VKA (%)	17 (32.1)	10 (9.7)	0.001
NOAC (%)	5 (9.4)	31 (30.1)	0.004
SAPT (%)	28 (52.8)	56 (53.3)	0.867
DAPT (%)	1 (1.9)	3 (2.9)	1.000

Data are presented as medians (25–75th quartiles) or as absolute numbers (percentages): AR: aortic regurgitation; AS: aortic stenosis; CAD: coronary artery disease; CKD: chronic kidney disease; COPD: chronic obstructive pulmonary disease; CVD: cerebrovascular disease; DAPT: dual antiplatelet therapy; ECMO: extracorporeal membrane oxygenation; IDDM: insulin-dependent diabetes mellitus; MAVD: mixed aortic valve disease; MI: myocardial infarction; NOAC: novel oral anticoagulants; PAD: peripheral artery disease; PTCA: percutaneous transluminal coronary angioplasty; PVL: paravalvular leakage; SAPT: single antiplatelet therapy; VKA: Vitamin K antagonist.

**Table 2:** Multivariable analysis: Heart Team decision.

	OR	95% CI	*p*-Value
Age (years)	1.061	1.020–1.104	0.004
IDDM	1.853	0.453–7.584	0.391
CAD	2.648	1.160–6.048	0.021
CNI	2.711	1.079–6.811	0.034
VKA	0.311	0.107–0.906	0.032
NOAC	1.599	0.462–1.599	0.459

The analysis was performed using binary logistic regression. OR: odds ratio; CI: confidence interval; IDDM: insulin-dependent diabetes mellitus; CAD: coronary heart disease; VKA: vitamin K antagonist; NOAC: new oral anticoagulant. Marked *p*-values are two-sided significant at least at the 0.05 level.

**Table 3:** Echocardiographic data.

	RS (n = 53)	ViV (n = 103)	*p*-Value
Before secondary intervention			
LVEF (%)			0.994
≥50	41 (1.2)	77 (74.7)	
31–49%	10 (18.8)	21 (20.3)	
≤30	1 (1.8)	5 (4.8)	
Aortic stenosis (%)			0.010
low to mild	14 (26.4)	6 (5.8)	
moderate to severe	38 (71.6)	90 (87.3)	
Aortic regurgitation			0.078
low to mild (%)	25 (47.1)	64 (62.1)	
moderate to severe (%)	28 (52.8)	32 (31.0)	
Mitral insufficiency			0.595
low to mild (%)	37 (69.8)	76 (73.7)	
moderate to severe (%)	15 (28.3)	20 (19.4)	
Tricuspid insufficiency (%)			0.115
moderate to severe (%)	3 (5.6)	19 (18.4)	
Pulmonary hypertension (%)	12 (22.6)	13 (12.6)	0.113
AV prosthesis PG max (mmHg)	73 (57–96)	61 (45–73)	0.694
AV prosthesis PG mean (mmHg)	45 (33–67)	37 (26–45)	0.391
AV prosthesis PVL (%)	7 (13.2)	9 (8.7)	0.364
EOA (cm^2^)	0.7 (0.5–0.8)	0.7 (0.6–0.9)	0.747
After secondary intervention			
PG max (mmHg)	18 (10–30)	26 (19–38)	<0.001
PG mean (mmHg)	9 (6–15)	15 (9–21)	<0.001
PG mean ≥ 20 mmHg (%)	1 (1.8)	25 (24.2)	<0.001
PVL (%)	1 (1.9)	23 (25.3)	0.013

Data are presented as medians (25–75th quartiles) or as absolute numbers (percentages): AV: aortic valve; LVEF: left ventricular ejection fraction; EOA: effective orifice area; PG: pressure gradient; PVL: paravalvular leakage.

**Table 4:** Details of surgery/intervention.

	RS (n = 53)	ViV (n = 103)
Bypass time (min)	156 (119–211)	-
Cross-clamp time (min)	103 (82–141)	-
Concomitant procedures (%)	33 (62.2)	16 (15.5)
Aortic root enlargement (%)	12 (22.6)	-
Aortic root replacement (%)	3 (5.7)	-
CABG surgery (%)	7 (13.2)	-
Mitral valve reconstruction (%)	1 (1.9)	-
Mitral valve replacement (%)	13 (24.5)	-
Tricuspid valve replacement (%)	2 (3.8)	-
PTCA/Stenting (%)	-	16 (15.5)
Valve prosthesis		
Biological (%)	40 (75.4)	103 (100)
Self-expandable TAVR		71 (68.9)
Balloon-expandable TAVR		32 (31.1)
Mechanical (%)	13 (24.5)	-

Data are presented as medians (25–75th quartiles) or as absolute numbers (percentages). CABG: coronary artery bypass grafting; PTCA: percutaneous transluminal coronary angioplasty; TAVR: transcatheter aortic valve replacement.

**Table 5:** (**a**) Echocardiographic data based on valve type. (**b**) Echocardiographic data based on valve type.

(**a**)			
	**Bioprosthetic (n = 40)**	**Mechanical (n = 13)**	***p*-Value**
PG max (mmHg)	18 (11–20)	21 (13–30)	0.629
PG mean (mmHg)	9 (6–11)	13 (8–17)	0.577
PVL (%)	1 (3.8)	0 (0.0)	1.000
(**b**)			
	**SE (n = 71)**	**BE (n = 32)**	***p*-Value**
PG max (mmHg)	22 (17–28)	28 (24–25)	0.073
PG mean (mmHg)	13 (9–21)	16 (13–21)	0.153
PVL (%)	20 (32.8)	3 (10.0)	0.021

Data are presented as medians (25–75th quartiles) or as absolute numbers (percentages). PG: pressure gradient; PVL: paravalvular leackage. SE: self-expandable; BE: balloon-expandable; Marked *p*-values are two-sided significant at least at the 0.05 level.

**Table 6:** Postoperative/postinterventional morbidities.

	RS (n = 53)	ViV (n = 103)	*p*-Value
Cardiovascular morbidities			
CPR (%)	0 (0.0)	6 (5.8)	0.096
Coronary obstruction (%)	0 (0.0)	3 (2.9)	0.551
Myocardial infarction (%)	0 (0.0)	2 (1.9)	0.548
Atrial fibrillation (%)	8 (15.1)	5 (4.9)	0.036
Arrythmia			
AV—block (%)	4 (7.5)	8 (2.6)	1.000
Bundle branch block (%)	5 (9.4)	21 (20.4)	0.112
Pacemaker implantation (%)	3 (5.7)	6 (5.8)	1.000
Other			
Bleeding (%)	5 (9.4)	9 (8.7)	1.000
Blood transfusion (%)	38 (71.6)	5 (4.8)	<0.001
Re-explorative surgery (%)	6 (11.3)	0 (0.0)	0.001
Adverse cerebrovascular events (%)	0 (0.0)	5 (4.9)	0.167
Sepsis (%)	5 (9.4)	3 (2.9)	0.122
Pneumonia (%)	7 (13.2)	5 (4.9)	0.108
Surgical site infection (%)	5 (9.4)	1 (1.0)	0.018
Groin complication (%)	1 (1.9)	8 (7.8)	0.168
Organ support			
Hemodialysis	7 (13.2)	2 (1.9)	0.008
IABP	1 (1.9)	0 (0.0)	0.340
ECMO	2 (3.8)	3 (2.9)	1.000

Data are presented as medians (25–75th quartiles) or as absolute numbers (percentages). AV—block: atrioventricular block; CPR: cardiopulmonary resuscitation; ECMO: extracorporeal membrane oxygenation; IABP: intra-aortic balloon pump.

**Table 7:** Postoperative/postinterventional outcomes.

	RS (n = 53)	ViV (n = 103)	*p*-Value
Length of ICU stay (days)	3 (3–5)	1 (1–3)	<0.001
Length of hospital stay (days)	15 (13–20)	11 (8–14)	<0.001
Discharge			<0.001
Cardiac rehabilitation (%)	43 (81.1)	41 (39.8)	
Inpatient care (%)	4 (7.54)	14 (13.5)	
Home (%)	3 (5.66)	44 (42.7)	
VARC-3 Early Safety (%)	43 (81.1)	90 (87.3)	0.343
30-day mortality (%)	3 (5.7)	3 (2.9)	0.409
Myocardial infarction (%)	0 (0.0%)	1 (0.9%)	1.000
New pacemaker implantation (%)	3 (5.6%)	6 (5.8%)	1.000
Hospital admission for cardiac reasons (%)	8 (15.1%)	11 (10.7%)	0.801
Redo aortic valve surgery (%)	0 (0.0%)	1 (0.9%)	1.000
Major bleeding (%)	1 (1.9%)	3 (2.9%)	1.000
Ischemic stroke (%)	3 (5.7%)	2 (1.9%)	0.370

Data are presented as medians (25–75th quartiles), as mean ± standard deviation, or as absolute numbers (percentages). VARC: valve academic research consortium.

**Table 8:** Postoperative/postinterventional morbidities and outcomes—subgroup analysis.

	RSs (n = 29)	ViV (n = 103)	*p*-Value
Cardiovascular morbidities			
CPR (%)	0 (0.0)	6 (5.8)	0.338
Coronary obstruction (%)	0 (0.0)	3 (2.9)	0.551
Myocardial infarction (%)	0 (0.0)	2 (1.9)	0.548
Atrial fibrillation (%)	5 (17.2)	5 (4.9)	0.041
Arrythmia			
AV—block (%)	2 (6.9)	8 (2.6)	1.000
Bundle branch block (%)	3 (10.3)	21 (20.4)	0.282
Pacemaker implantation (%)	1 (3.4)	6 (5.8)	1.000
Other			
Bleeding (%)	1 (3.4)	9 (8.7)	0.691
Blood transfusion (%)	18 (62.1)	5 (4.8)	<0.001
Re-explorative surgery (%)	1 (3.4)	0 (0.0)	0.220
Adverse cerebrovascular events (%)	0 (0.0)	5 (4.9)	0.585
Sepsis (%)	2 (6.9)	3 (2.9)	0.302
Pneumonia (%)	1 (3.4)	5 (4.9)	1.000
Surgical site infection (%)	0 (0.0)	1 (1.0)	1.000
Groin complication (%)	0 (0.0)	8 (7.8)	0.199
Organ support			
Hemodialysis (%)	2 (6.9)	2 (1.9)	0.121
IABP (%)	0 (0.0)	0 (0.0)	-
ECMO (%)	0 (0.0)	3 (2.9)	1.000
Outcomes			
Length of ICU stay (days)	2 (2–3)	1 (1–3)	0.011
Length of hospital stay (days)	15 (12–21)	11 (8–14)	<0.001
VARC-3 Early Safety (%)	26 (89.7)	90 (87.3)	1.000
30-day mortality (%)	1 (3.4)	3 (2.9)	1.000

Data are presented as medians (25–75th quartiles) or as absolute numbers (percentages). RSs: Redo-SAVR subgroup; AV—block: atrioventricular block; CPR: cardiopulmonary resuscitation; ECMO: extracorporeal membrane oxygenation; IABP: intra-aortic balloon pump. VARC: valve academic research consortium.

## Data Availability

The data presented in this study are available on request from the corresponding author due to national data safety regulations.
